# The apoptotic and anti‐proliferative effects of Neosetophomone B in T‐cell acute lymphoblastic leukaemia via PI3K/AKT/mTOR pathway inhibition

**DOI:** 10.1111/cpr.13773

**Published:** 2024-11-14

**Authors:** Shilpa Kuttikrishnan, Abdul W. Ansari, Muhammad Suleman, Fareed Ahmad, Kirti S. Prabhu, Tamam El‐Elimat, Feras Q. Alali, Ammira S. Al Shabeeb Akil, Ajaz A. Bhat, Maysaloun Merhi, Said Dermime, Martin Steinhoff, Shahab Uddin

**Affiliations:** ^1^ Translational Research Institute, Academic Health System Hamad Medical Corporation Doha Qatar; ^2^ College of Pharmacy, QU Health Qatar University Doha Qatar; ^3^ Dermatology Institute, Academic Health System Hamad Medical Corporation Doha Qatar; ^4^ Laboratory of Animal Research Center Qatar University Doha Qatar; ^5^ Department of Medicinal Chemistry and Pharmacognosy, Faculty of Pharmacy Jordan University of Science and Technology Irbid Qatar; ^6^ Department of Human Genetics‐Precision Medicine in Diabetes, Obesity and Cancer Program Sidra Medicine Doha Qatar; ^7^ Translational Cancer Research Facility, National Center for Cancer Care and Research Hamad Medical Corporation Doha Qatar; ^8^ College of Health Sciences Qatar University Doha Qatar; ^9^ Department of Dermatology & Venereology Hamad Medical Corporation Doha Qatar; ^10^ Department of Medicine Weill Cornell Medicine‐Qatar Doha Qatar; ^11^ College of Medicine Qatar University Doha Qatar; ^12^ College of Health and Life Sciences Hamad Bin Khalifa University Doha Qatar; ^13^ Department of Medicine Weill Cornell Medicine New York New York USA

## Abstract

The phosphatidylinositol 3‐kinase/Protein Kinase B/mammalian target of rapamycin (PI3K/AKT/mTOR) signalling pathway is pivotal in various cancers, including T‐cell acute lymphoblastic leukaemia (T‐ALL), a particularly aggressive type of leukaemia. This study investigates the effects of Neosetophomone B (NSP‐B), a meroterpenoid fungal metabolite, on T‐ALL cell lines, focusing on its anti‐cancer mechanisms and therapeutic potential. NSP‐B significantly inhibited the proliferation of T‐ALL cells by inducing G0/G1 cell cycle arrest and promoting caspase‐dependent apoptosis. Additionally, NSP‐B led to the dephosphorylation and subsequent inactivation of the PI3K/AKT/mTOR signalling pathway, a critical pathway in cell survival and growth. Molecular docking studies revealed a strong binding affinity of NSP‐B to the active site of AKT, primarily involving key residues crucial for its activity. Interestingly, NSP‐B treatment also induced apoptosis and significantly reduced proliferation in phytohemagglutinin‐activated primary human CD3^+^ T cells, accompanied by a G0/G1 cell cycle arrest. Importantly, NSP‐B did not affect normal primary T cells, indicating a degree of selectivity in its action, targeting only T‐ALL cells and activated T cells. In conclusion, our findings highlight the potential of NSP‐B as a novel therapeutic agent for T‐ALL, specifically targeting the aberrantly activated PI3K/AKT/mTOR pathway and being selective in action. These results provide a strong basis for further investigation into NSP‐B's anti‐cancer properties and potential application in T‐ALL clinical therapies.

## Graphical abstract

Anticancer mechanism of NSP‐B in T‐ALL.

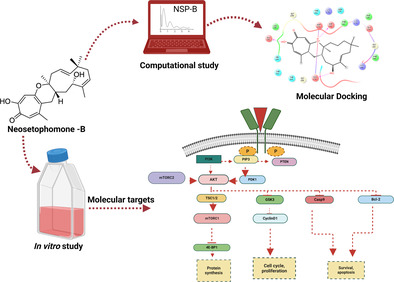

## 1 INTRODUCTION

T‐cell acute lymphoblastic leukaemia (T‐ALL), a devastating hematologic malignancy, arises from the malignant transformation of T lymphocyte progenitors. Characterized by an accumulation of immature, undifferentiated thymocytes, T‐ALL exhibits a complex genetic landscape marked by a plethora of mutations. These genetic aberrations disrupt key survival signalling pathways, contributing to the disease's notoriously poor prognosis, especially in recurrent cases.^1^ Notably, T‐ALL is associated with the production of numerous abnormal leukaemia cells, and these malignant T cells often infiltrate the bone marrow, impeding the normal production of red blood cells, platelets and white blood cells.^2^ Recent advancements, such as improved glucocorticoids and asparaginase therapies, have significantly enhanced patient outcomes.^3^, ^4^ However, treating adult T‐ALL remains particularly challenging due to the emergence of chemotherapy resistance and the high incidence of refractory relapse,^5^ underscoring an urgent need for novel treatment approaches.

Natural products and their derivatives, renowned for their structural diversity and versatile pharmacological properties, have emerged as potential keystones in cancer therapy development.^6^ Neosetophomone B (NSP‐B), a meroterpenoid fungal secondary metabolite derived from *Neosetophoma* sp., has demonstrated notable cytotoxicity against leukaemias, multiple myeloma and breast and ovarian cancer cell lines at micromolar concentrations.^7^ Recent findings indicate that NSP‐B induces cell death in leukaemic cells by inhibiting the AKT/SKP2 axis and activating mitochondrial and caspase‐dependent signalling pathways.^8^

The mammalian target of rapamycin (mTOR), a serine–threonine kinase, plays a pivotal role in regulating cell survival and proliferation. Dysregulation of the mTOR signalling pathway is common in various human diseases, including cancers.^9^, ^10^, ^11^ mTOR functions through two distinct complexes, mTORC1 and mTORC2.^12^ mTORC1, sensitive to changes in oxygen levels, growth factors and nutrient status.^13^, ^14^, ^15^ Conversely, mTORC2, which is rapamycin‐resistant and less influenced by nutrient status, primarily regulates cancer cell growth and proliferation and is key in AKT activation at Ser473.^12^, ^15^, ^16^, ^17^ Notably, aberrant activation of the AKT pathway is a hallmark in many human malignancies,^18^, ^19^, ^20^ and this is particularly relevant in T‐ALL, where the constitutive activation of the phosphatidylinositol 3‐kinase/Protein Kinase B/mTOR (PI3K/AKT/mTOR) pathway has been observed in 70%–85% of patients, often correlating with poor clinical outcomes.^21^, ^22^, ^23^ Thus, targeting the AKT/mTOR signalling pathway presents a compelling strategy in cancer therapeutics.

In this study, we investigate the antitumor potential of NSP‐B in T‐ALL cell lines and in vitro‐generated primary human T‐cell phytohemagglutinin (PHA) blasts. We focus on evaluating the effects of NSP‐B on cell proliferation, cell cycle regulation and the suppression of the PI3K/AKT/mTOR signalling pathway.

## 2 MATERIALS AND METHODS

### 2.1 Isolation of NSP‐B from fungi

NSP‐B was isolated from *Neosetophoma* sp. [strain MSX50044] as reported by El‐Elimat et al.^7^ The purity of NSP‐B was evaluated by UPLC and found to be >97%.^7^

### 2.2 Reagents and antibodies

Cell Counting Kit‐8 (CCK‐8), methanol and dimethylsulphoxide were obtained from Sigma‐Aldrich (St. Louis, MO, USA). Antibodies against caspase‐3, poly (ADP‐ribose) polymerase (PARP), Bcl2, Bid, cleaved caspase‐8, P‐AKT, AKT, P‐MTOR, MTOR, P‐4EBP1, 4EBP1, P‐GSK3α/β, GSK3α/β, CDK‐4,6, Cyclin D1 and GAPDH were purchased from Cell Signaling Technologies (Beverly, MA, USA). HSP60 and BAX from Santa Cruz Biotechnolog Inc. (CA, USA). Live/Dead assay kit, RPMI 1640 medium, foetal bovine serum and penicillin/streptomycin cocktail were obtained from Life Technologies, Inc. (Carlsbad, CA).

### 2.3 Cell lines

The following T‐cell lines; Jurkat (TIB‐152), Molt 4 (CRL‐1582) and Molt 3 (CRL‐1552) were derived from the patient with T‐ALL and were purchased from ATCC (Manassas, Virginia, USA) and cultured in RPMI 1640 media supplemented with 10% foetal bovine serum, 100 U/mL penicillin and 100 U/mL streptomycin. The cells were maintained at 37°C in a humidified incubator with 5% CO_2_.^24^

### 2.4 Cell viability assay

To assess the anti‐proliferative effects of NSP‐B on T‐ALL cells, we utilized the CCK‐8 colorimetric assay.^25^

### 2.5 Live and dead cell viability assay

Post‐treatment with NSP‐B, Jurkat, Molt 4 and Molt 3 cells were stained using a live/dead viability assay. The stained cells were incubated in the dark for 15–30 min at room temperature. Cellular morphology was observed under 20× magnification using an Invitrogen EVOS FLoid Cell Imaging System.^25^

### 2.6 Measurement of apoptosis

Following NSP‐B treatment, cells were stained with Annexin V‐FITC and propidium iodide (PI) for apoptosis assessment. Flow cytometry using the BD LSR Fortessa analyzer (BD Biosciences) categorized the cells into necrotic, early apoptotic, late apoptotic or live based on Annexin FITC and PI staining. The total percentage of apoptosis was calculated by summing the percentages of early and late apoptotic cells.^26^, ^27^

### 2.7 Cell cycle analysis

Cell cycle distribution was analysed in Jurkat and Molt 4 cells post 48‐h treatment with NSP‐B. Cells were stained with Hoechst 33342 fluorescent dye and incubated for 30 min at 37°C. Flow cytometry using the BD LSR Fortessa analyzer (BD Biosciences) was utilized to determine the cell cycle phases.^25^

### 2.8 Flow cytometric analysis of mTOR activity

To assess mTOR activity, intracellular phosphorylated S6 ribosomal protein (pS6) levels were detected as described previously.^28^ Jurkat cells treated with NSP‐B were fixed, permeabilized and stained with anti‐pS6RP‐Alexa Fluor 647 antibody (Ser235/236, clone 2F9, Cell Signaling Technologies). The eBiosciences FoxP3/Transcription factor staining kit (ThermoFisher, USA) was used for cell preparation. Flow cytometry acquisition was performed to quantify pS6 levels, indicative of mTOR activity.

### 2.9 Human T‐cell isolation, PHA activation, apoptosis and cell cycle analyses

Primary human CD3^+^ T cells were enriched from peripheral blood mononuclear cells isolated from buffy coats of healthy volunteers using Ficoll‐Paque density gradient centrifugation as described previously.^28^ Afterwards, T cells were isolated using EasySep Human T Cell Enrichment kit (Stem Cell Technologies, USA) as per manufacturer's instruction. The purity of T cells was routinely tested using surface staining with BUV737‐labelled anti‐CD3 antibody (BD Biosciences, USA). Flow cytometry analysis revealed a purity of more than 95%.

Briefly, 1 × 10^6^ purified T cells were stimulated with PHA‐L (Sigma Aldrich) at a concentration of 5 μg/mL in the presence or absence of NSP‐B in a 24‐well culture plate. For apoptosis assay, cells were harvested after 24 h, stained with annexin V and PI and analysed by flow cytometry as described above. For cell cycle analysis, purified T cells were stimulated for 3 days to generate a T‐cell blast in the presence or absence of NSP‐B. Cells were harvested, labelled with PI as described above and analysed using flow cytometry. Unstimulated T cells served as normal control cells which were also treated in the presence or absence of NSP‐B.

### 2.10 Molecular docking of NSP‐B with the AKT

To explore the interaction between NSP‐B and AKT, we employed a molecular docking approach. The crystal structure of AKT (ID: 4EKL) was retrieved from the Protein Data Bank (https://www.rcsb.org/structure/5nt1).^29^ Following this, we conducted refinement procedures, which included adding hydrogen atoms and minimizing the protein structure using Chimera.^30^, ^31^ The 3D structure of NSP‐B, retrieved from PubChem (ID: 146683131) at https://pubchem.ncbi.nlm.nih.gov/compound/Neosetophomone-B, underwent minimization using the MMFFx force field. For the docking process, we used induced‐fit docking (IFD) with AutoDockFR, leveraging force fields like AMBER or CHARMM, simulation protocols such as molecular dynamics (MD), and scoring functions like AMBER scoring or force‐field‐based scoring for IFD simulations. Default parameters were applied for IFD docking, enabling receptor flexibility and accommodating covalent docking.^32^ The resulting complex was then subjected to visual analysis using PyMOL and Schrodinger Maestro (utilizing the free academic version for visualization). Subsequent molecular simulations were conducted to further validate the findings.^33^

### 2.11 Molecular dynamic simulation analysis of NSP‐B‐AKT complex

MD simulations were initiated by preparing coordinate and topology files using the “tLeap” module in AMBER21.^34^, ^35^ MD simulation was conducted in several stages, including energy minimization, heating, equilibration and production. Each system was solvated, and ions were introduced to balance the charge. While antechamber and parmchk2 were used to produce the initial topology and frcmod file while the GAFF2 force field was used to parameterize the ligand molecule. After neutralization, a two‐step energy minimization protocol was applied to resolve any bad clashes. This consisted of 9000 cycles, with the first 6000 cycles using the steepest descent method for minimization,^36^ followed by 3000 cycles employing the conjugate gradient method.^37^ After the minimization, the system was equilibrated and gradually heated to 310 K under a constant pressure of 1 atm. The equilibration process included positional restraint, gradual heating and unrestrained equilibration. The SHAKE algorithm maintained covalent bond lengths and angles, and pressure control was maintained with a barostat. After equilibration, a production simulation of 100 ns was performed for each system using a MD algorithm, either under NPT or NVT ensemble conditions.^38^

### 2.12 Post‐simulation analysis

The trajectory resulting from simulation production was analysed through the application of CPPTRAJ or PTRAJ modules.^39^ Key metrics, including root mean square deviation (RMSD), root mean square fluctuation (RMSF) and hydrogen bonding, were computed for each system.^40^, ^41^, ^42^ RMSD, a measure of variance between the original and superimposed structures, is calculated using the mathematical equation:

(1) $$\text{RMSD}=\sqrt{\frac{\sum d^{2}i=1}{N_{\text{atoms}}}}.$$

Furthermore, the assessment of RMSF involves utilizing the *B*‐factor to gauge the flexibility of individual protein residues. The mathematical expression for RMSF is as follows:

(2) $$\text{Thermal factor or}\;B‐\text{factor}=\left[\frac{\left(8\pi **2\right)}{3}\right]\left(\mathrm{msf}\right).$$

### 2.13 Binding free energy calculation

The molecular mechanics (MM) generalized Born surface area method was employed to estimate the end‐point total binding free energy, a commonly used and dependable approach for assessing the binding affinity of protein‐ligand complexes.^33^ The MMPBSA.py script was employed to select stable frames from the simulation trajectories for the subsequent free energy calculations.^43^ The computation of the total binding free energy involved determining the difference between the free energy of the complex and the summation of the free energies of the individual protein and ligand components, as illustrated in the following equation^44^:

(3) $$\Delta G\text{bind}=\Delta G\text{complex}-\left(\Delta G\text{receptor}+\Delta G\text{ligand}\right).$$

The free energy for each component was determined by aggregating various energy terms, encompassing bonded, electrostatic, Van der Waals, as well as polar and non‐polar contributions. This calculation is represented by the following equation^44^:

(iv) G=Gbond+Gelectrostatic+GvdW+Gpolar+Gnon‐polar.

## 3 RESULTS

### 3.1 NSP‐B suppresses proliferation and induces apoptotic cell death in T‐ALL cell lines

**FIGURE 1:**
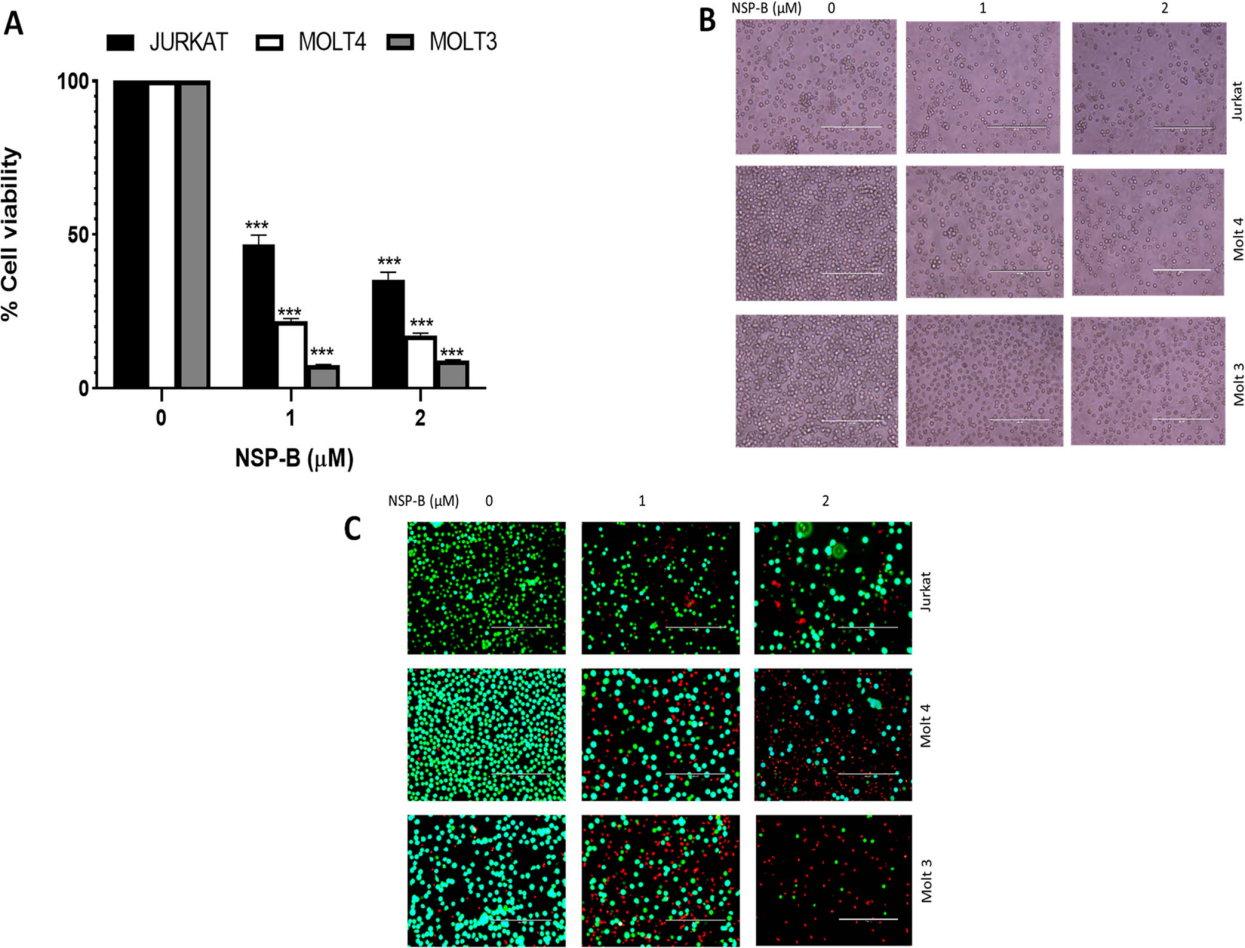
Effect of Neosetophomone B (NSP‐B) on T‐cell viability. (A) NSP‐B's inhibitory effect on T‐cell viability: This panel presents the results of a cell viability assay conducted on Jurkat, Molt 4 and Molt 3 cells. Cells were incubated with NSP‐B at concentrations of 0, 1 and 2 μM for 48 h. Viability was assessed using the Cell Counting Kit‐8 assay, as detailed in the Section 2. The graph depicts the average cell viability (mean ± SD) from three independent experiments. A statistically significant reduction in cell viability (***p < 0.001) was observed at higher concentrations of NSP‐B. (B, C) Induction of cell death by NSP‐B in T‐cell lines: This section shows the morphological changes and cell death induction in Jurkat, Molt 4 and Molt 3 cells treated with 1 and 2 μM NSP‐B for 48 h. Morphological observations were made under a microscope at 20× magnification. Following this, cells were stained with a live/dead reagent and further examined under a fluorescent microscope (20× magnification; scale bar: 200 μm).

Our initial investigations focused on assessing the anti‐proliferative effects of NSP‐B on T‐ALL cells. We treated a panel of T‐ALL cell lines, including Jurkat, Molt‐4 and Molt‐3, with 1 and 2 μM NSP‐B for 48 h. Post‐treatment cell viability was evaluated using the CCK‐8. A dose‐dependent decrease in cell proliferation was observed across all cell lines, as illustrated in Figure 1A. This growth inhibition by NSP‐B was statistically significant at both concentrations. Morphological analysis complemented these findings, revealing the presence of apoptotic bodies in response to NSP‐B treatment in all tested cell lines (Figure 1B).

To determine if the reduced cell viability was attributable to cell death, we performed a live/dead assay. Following NSP‐B treatment, Jurkat, Molt‐4 and Molt‐3 cells were stained using a live/dead assay kit. As shown in Figure 1C, there was a notable increase in dead cells in a dose‐dependent manner, indicating NSP‐B's efficacy in inducing cell death.

**FIGURE 2:**
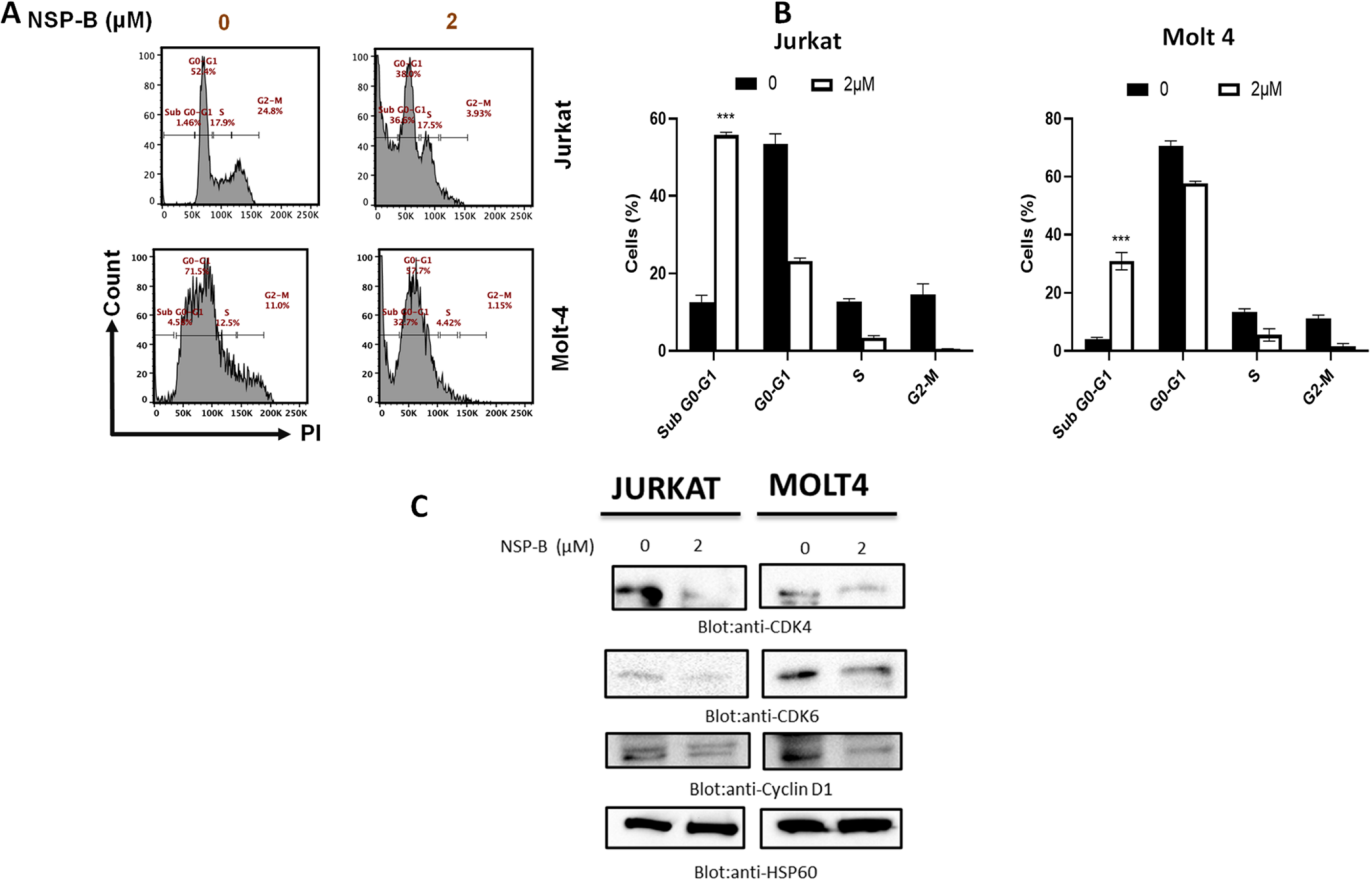
NSP‐B's effect on cell cycle and cyclin/CDK expression in T cells. (A, B) Effect of NSP‐B on cell cycle distribution. Jurkat and Molt 4 cells were treated with NSP‐B, and subsequent alterations in cell cycle phases were analysed using flow cytometry. The graph shows the mean ± SD from three independent experiments, highlighting significant alterations (***p < 0.001). (C) NSP‐B's Effect on Cyclin and CDKs: This panel illustrates the impact of NSP‐B on key cell cycle regulators. Jurkat and Molt 4 cells treated with varying NSP‐B concentrations showed downregulation of CDK‐4, CDK‐6 and cyclin D1, as evidenced by immunoblotting. HSP60 served as a loading control. NSP‐B, Neosetophomone B; PI, propidium iodide.

Subsequently, we explored NSP‐B's impact on cell cycle regulation. Flow cytometry analysis revealed a significant accumulation of cells in the SubG0/G1 phase, indicative of apoptotic cell death, along with a corresponding decrease in other cell cycle phases after 48 h of NSP‐B treatment (Figure 2A,B). Moreover, NSP‐B treatment led to the downregulation of key cell cycle regulators, including cyclin D1 and cyclin‐dependent kinases CDK‐6 and CDK‐4 (Figure 2C), further corroborating its role in cell cycle arrest.

**FIGURE 3:**
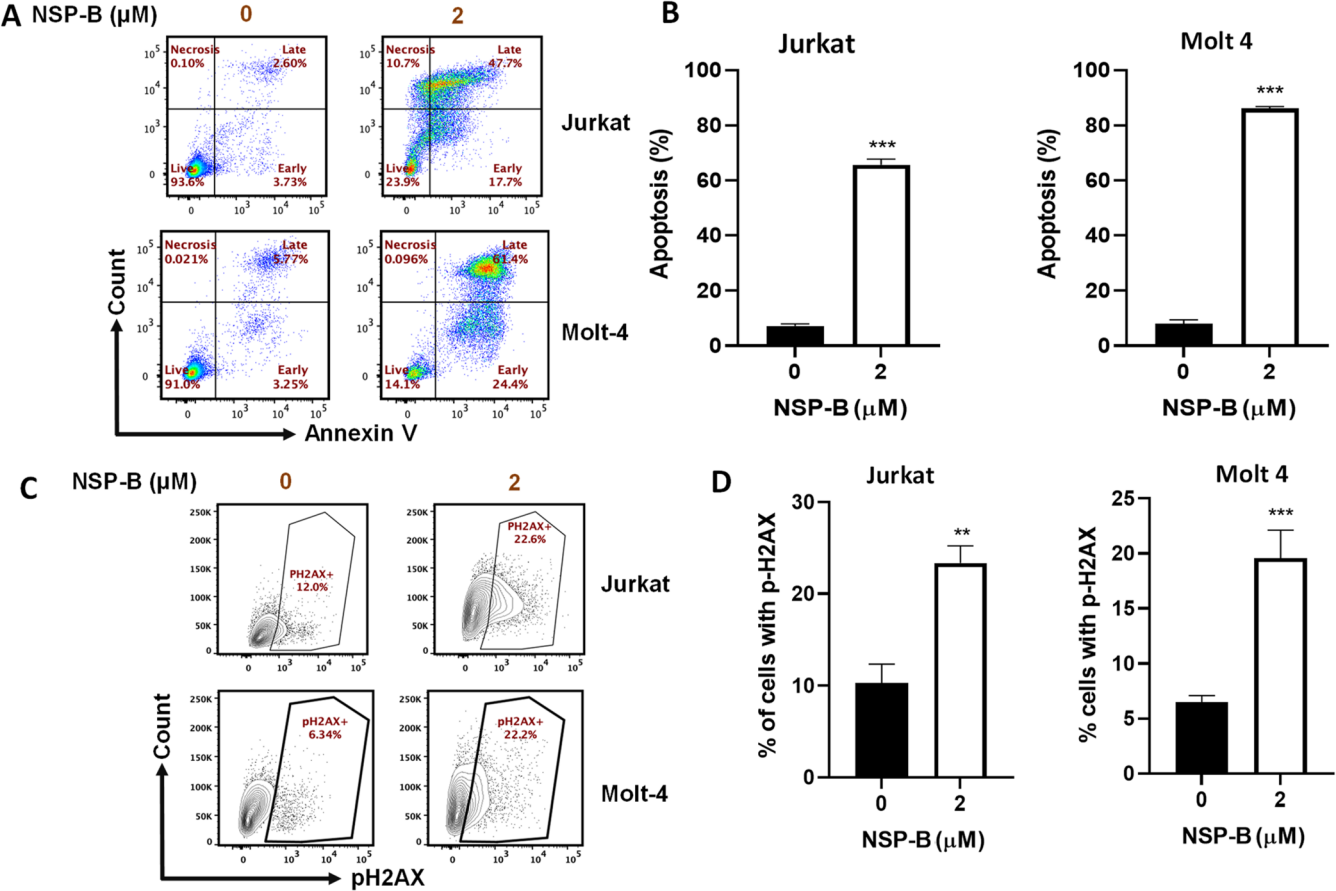
Neosetophomone B (NSP‐B)‐induced apoptosis and DNA damage in T‐cell lines. (A, B) NSP‐B‐mediated apoptosis in T cells: Demonstrated here is the proapoptotic effect of NSP‐B (1 and 2 μM) on Jurkat and Molt 4 cells. Apoptosis was quantified post‐staining with fluorescein‐conjugated Annexin‐V/PI and analysed via flow cytometry. The graph presents the mean ± SD of three independent experiments, with ***p < 0.001 indicating significant apoptotic induction. (C, D) NSP‐B‐Induced H2AX Phosphorylation: These panels display the levels of phosphorylated H2AX (pH2AX), a marker of DNA damage, in cells treated with NSP‐B. Flow cytometry data are summarized in the graphs, showing the mean ± SD from three independent experiments (**p < 0.01, ***p < 0.001).

Further confirming NSP‐B's role in inducing apoptosis, we performed Annexin V/PI dual staining. Jurkat and Molt‐4 cells treated with 1 and 2 μM NSP‐B for 48 h exhibited significant apoptosis, as compared to control cells, at both NSP‐B concentrations (Figure 3A,B), with statistical significance (*p* < 0.05).

Lastly, we observed a dose‐dependent increase in the expression of phosphorylated H2AX, a marker of double‐stranded DNA breaks, following NSP‐B treatment (Figure 3C,D). This finding underscores the potent DNA damage‐inducing capability of NSP‐B in T‐ALL cells, adding another dimension to its mechanism of action.

### 3.2 NSP‐B induces caspase‐mediated apoptotic signalling in T‐ALL cells

**FIGURE 4:**
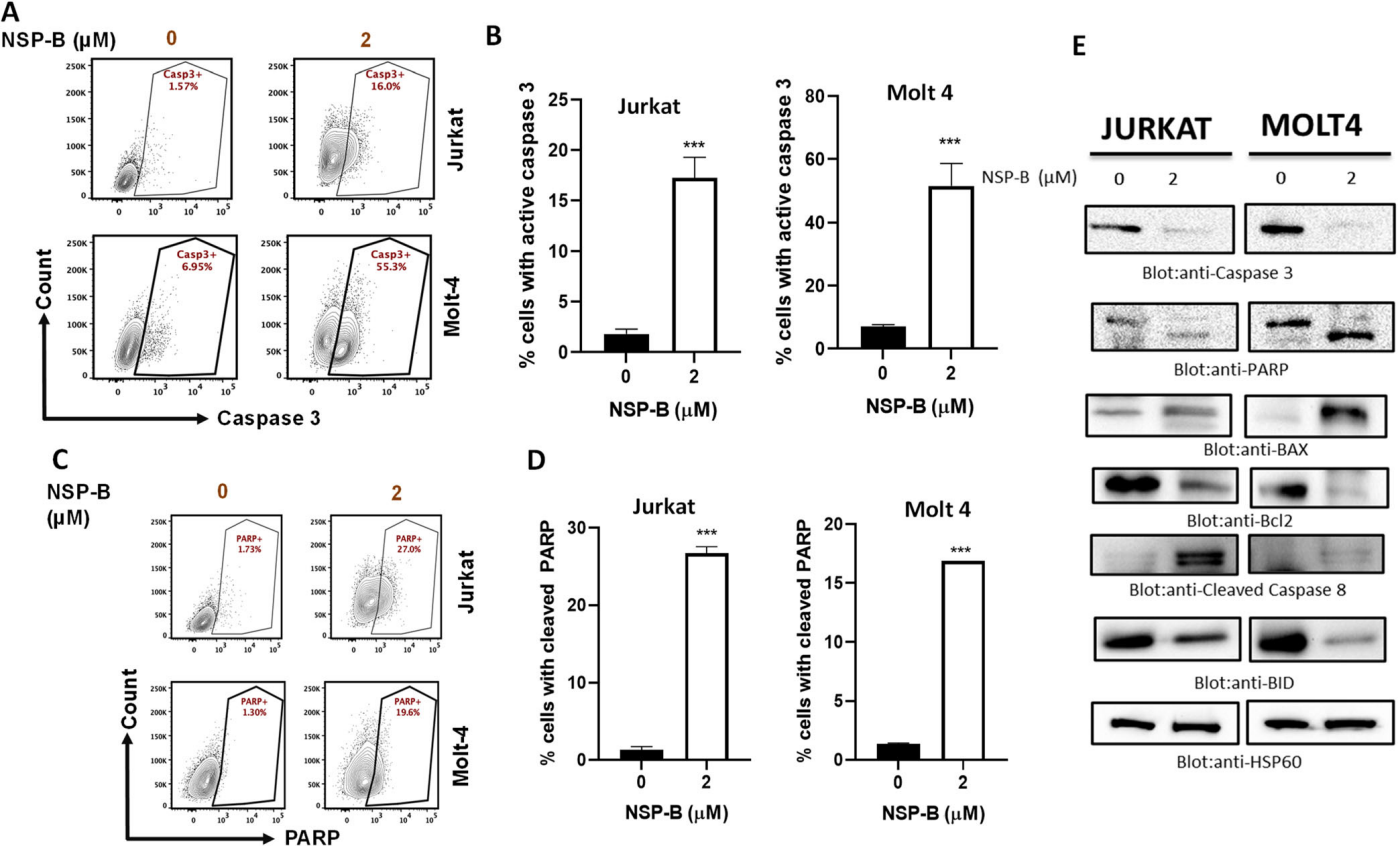
Neosetophomone B (NSP‐B) induces apoptotic pathways and caspase activation in T‐cell LINES. (A–D) Activation of Caspase Cascade by NSP‐B: This series of panels illustrates NSP‐B's role in triggering caspase‐3 activation and poly (ADP‐ribose) polymerase (PARP) cleavage, as determined by flow cytometry. Data are presented as mean ± SD from three independent experiments (***p < 0.001). (E) Effect on Apoptotic Protein Expression: Jurkat and Molt 4 cells treated with NSP‐B exhibited changes in the expression of caspase‐8, BID, Bcl2, Bax, caspase‐3, PARP and cleaved caspase 8. Proteins (50 μg) were separated via SDS–PAGE and probed with specific antibodies. HSP60 served as a loading control.

Recognizing the pivotal role of caspases in orchestrating the apoptotic response to a variety of stimuli,^45^, ^46^ To investigate this, we treated Jurkat and Molt4 cell lines with NSP‐B for 48 h and subsequently analysed cell lysates using immunoblotting techniques, targeting key apoptotic markers: caspase‐3, cleaved caspase‐3 and PARP. Our results, depicted in Figure 4A–E, reveal a marked activation of caspase‐9 and caspase‐3 cleavage in both T‐ALL cell lines following NSP‐B treatment. We investigated deeper to assess the influence of NSP‐B on the component of other caspase signalling. As shown in Figure 4E shows a notable reduction in the intensity of the pro‐caspase‐8 full‐length band in NSP‐B‐treated cells, suggesting the activation of caspase‐8 in both Jurkat and Molt4 lines. Additionally, the interplay between caspase‐8 and Bid during apoptosis was explored. Caspase‐8 is known to directly cleave Bid, a BH3‐only proapoptotic protein, facilitating its truncated form to trigger the Bax‐mediated mitochondrial apoptotic pathway.^47^, ^48^ Our findings indicate that NSP‐B treatment potentially promotes this cascade, leading to the activation of the intrinsic mitochondrial pathway.

### 3.3 NSP‐B induces apoptosis in T‐ALL cells via AKT/mTOR signalling pathway inhibition

**FIGURE 5:**
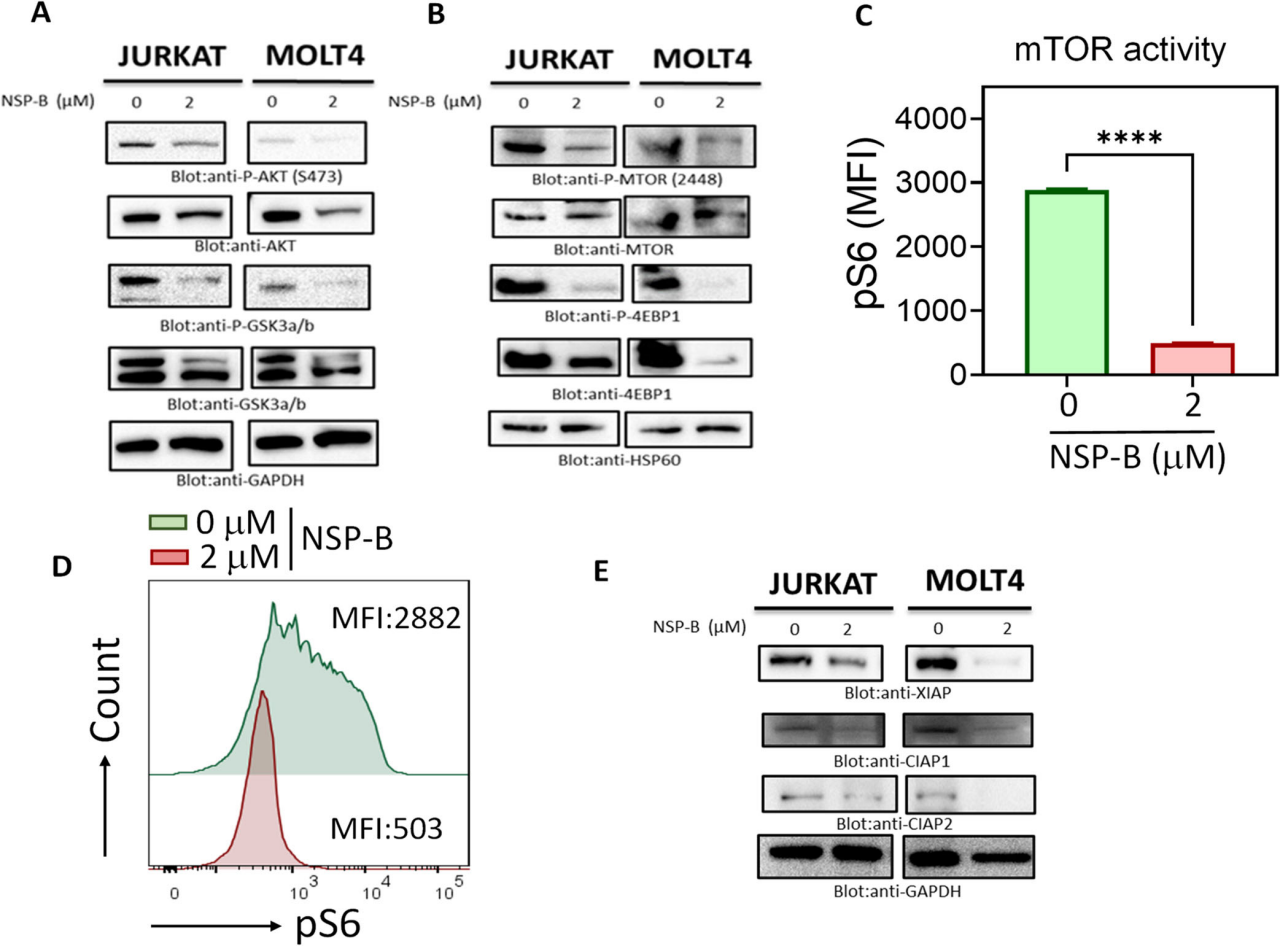
NSP‐B's effect on Protein Kinase B/mammalian target of rapamycin (AKT/MTOR) signalling and apoptotic inhibitors in T cells. (A, B, E) Modulation of AKT/MTOR Pathway by NSP‐B: Here, the impact of NSP‐B on AKT (Ser473), MTOR (S2448), 4EBP1 and GSK3α/β phosphorylation and the expression of apoptotic inhibitors (XIAP, c‐IAP1 and c‐IAP2) is shown. Proteins from treated cells were resolved by SDS–PAGE and analysed by immunoblotting using specific antibodies. GAPDH and HSP60 were used as loading controls. (C) mTOR Activity Assessment: This panel depicts the intracellular levels of phosphorylated S6 ribosomal protein (pS6), a downstream target of mTOR, in cells treated with or without 2 μM NSB‐P. Anti‐pS6 monoclonal antibody was used for intracellular staining, with FACS histograms showing the mean fluorescence intensity (MFI). (D) Statistical analysis of pS6 MFI: A representative plot illustrates the pS6 MFI (mean ± SD) from two independent experiments, each conducted in triplicates. Statistical significance was calculated using an unpaired *t*‐test (****p < 0.0001). NSP‐B, Neosetophomone B.

Having established the proapoptotic effects of NSP‐B on T‐ALL cells, our next endeavour was to elucidate the underlying molecular mechanisms. Specifically, we aimed to determine if the apoptotic induction by NSP‐B was mediated through the inactivation of the AKT/mTOR signalling pathway and its downstream targets. To this end, we treated Jurkat and Molt4 T‐ALL cell lines with 2 μM NSP‐B for a period of 48 h. Post‐treatment, we performed immunoblotting using antibodies targeting key components of the AKT/mTOR pathway. Our findings, illustrated in Figure 5A, demonstrated a notable inactivation of AKT in both cell lines following NSP‐B treatment. Furthermore, this treatment also led to the dephosphorylation of downstream targets, namely GSK3 α/β and 4E‐BP1, indicating a broad impact on the pathway.

Given the intricate relationship between mTOR and the AKT pathway and their established crosstalk in various cancer types,^49^, ^50^ we were particularly interested in examining the effect of NSP‐B on mTOR activity in T‐ALL cells. As depicted in Figure 5B, NSP‐B treatment resulted in the dephosphorylation of mTOR at Ser2448 in both cell lines. This effect was accompanied by a suppression in the expression of the translational repressor 4E‐BP1, a well‐known target of mTOR signalling.

To further validate the impact of NSP‐B on mTOR activity, we conducted intracellular pS6 staining and flow cytometric analysis. As presented in Figure 5C,D, the results showed a significant suppression in the phosphorylation levels of S6 in Jurkat cells treated with NSP‐B.

### 3.4 Modulation of IAP family proteins by NSP‐B in T‐ALL cell lines

In our pursuit to understand the mechanisms underlying NSP‐B‐induced cell death, we focused on the role of the inhibitors of apoptosis protein (IAP) family members. These proteins are crucial in dictating a cell's response to apoptotic triggers. To this end, we treated Jurkat and Molt4 T‐ALL cell lines with 2 μM of NSP‐B for a duration of 48 h. Our objective was to assess the impact of NSP‐B on the expression levels of key IAP family proteins: XIAP, cIAP1 and cIAP2.

Western blot analysis was employed to evaluate the expression of these proteins post‐treatment. As depicted in Figure 5E, the results revealed a significant downregulation of XIAP, cIAP1 and cIAP2 in both cell lines following NSP‐B treatment. This reduction in the levels of these critical IAP proteins suggests a direct influence of NSP‐B on the apoptotic machinery of T‐ALL cells.

**FIGURE 6:**
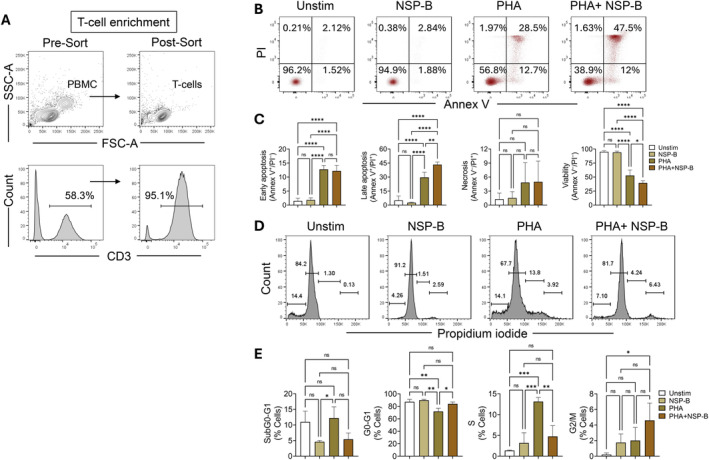
Effects of Neosetophomone B (NSP‐B) on primary human T cells. (A) Primary human CD3^+^ T‐cell enrichment. Representative FACS plots show the purity of CD3^+^ T cells pre‐ and post‐sorting, which is more than 95%. (B) NSP‐B induces cell death in phytohemagglutinin (PHA)‐activated T cells. Purified T cells were hyperactivated with PHA in the presence or absence of NSP‐B and assessed for cell death using flow cytometry. Representative FACS plots showing the frequency of cells in early (annex V^+^/PI^−^), late (annex V^+^/PI^+^) apoptosis, necrosis (annex V^−^/PI^+^) and viability (annex V^−^/PI^−^). Unstimulated (unstim) (medium only) primary T cells with or without NSP‐B treatment served as controls. (C) The statistical plot represents the mean ± SD of *n* = 4 biological replicates. (D) Effect of NSP‐B on cell cycle of T cell PHA blast. Cells were treated with or without NSP‐B for 3 days and analysed for cell cycle using propidium iodide (PI). Cells treated with NSP‐B served as controls. Representative FACS histograms show various phases of the cell cycle. (E) The statistical plot represents mean ± SD of *n* = 3 biological replicates. *p < 0.05; **p < 0.01; ***p < 0.001; ****p < 0.0001, ns stands for non‐significant.

Given the apoptotic induction of T‐ALL cell lines following NSP‐B treatment, we extended our investigation further to assess the potential impact of NSP‐B on primary human CD3^+^ T cells. Healthy donor T cells with purity above 95% (Figure 6A) were hyperactivated using mitogen PHA and examined for cell death. Flow cytometric data revealed significantly increased apoptosis in NSB‐P treated compared to untreated cells (Figure 6B,C). Interestingly, this effect was more pronounced with respect to late apoptosis as higher frequency of Annex V^+^/PI^+^ cells was seen in NSP‐B treated samples. Moreover, increased apoptosis is clearly reflected as reduced viability (Annex V^−^/PI^−^) of compound‐treated cells. However, our study found that NSP‐B did not exhibit any significant effects on unstimulated normal T cells. This lack of impact suggests that NSP‐B demonstrates a notable degree of selectivity, preferentially targeting cancerous cells while sparing normal, non‐cancerous T cells. This selectivity is particularly important, as it indicates the potential for NSP‐B to minimize adverse effects on healthy cells, making it a promising candidate for therapeutic development in cancer treatment.

### 3.5 NSP‐B alters the cell cycle by promoting primary T cells' G0/G1 arrest

As described above, NSP‐B significantly alters the cell cycle in T‐ALL cell lines. To investigate this effect in primary human T cells, a PHA blast was generated as described previously.^51^ Briefly, cells were stimulated with PHA in the presence or absence of NSP‐B for 3 days and subjected to cell cycle analysis using a flow cytometer. Unlike untreated cells, NSP‐B‐treated cells exhibited more cells at the G0/G1 stage (Figure 6D,E). Notably, NSP‐B inhibited cell proliferation as S‐phase displays lower DNA content than cells treated with PHA only. However, we could not see a significant change in the G2/M phase of the cell cycle, though there is a sort of trend towards G2/M arrest.

### 3.6 Bonding network analysis of NSPB with AKT

**FIGURE 7:**
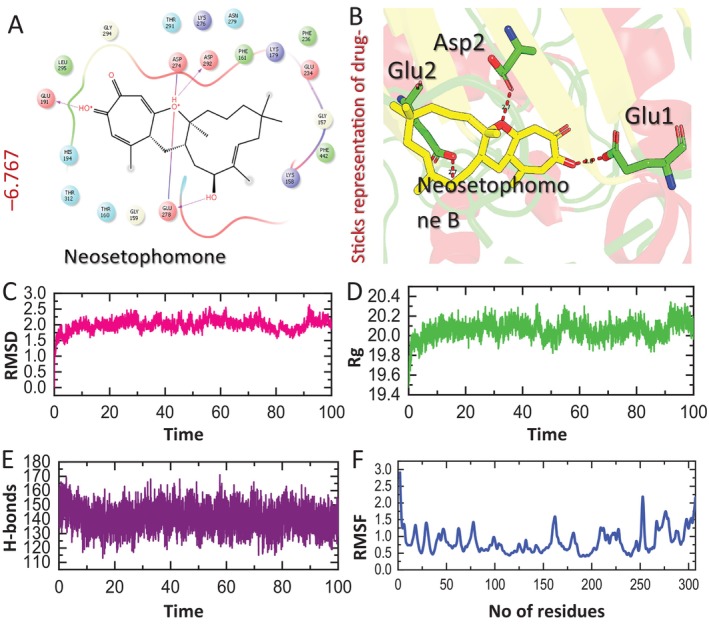
Bonding network and dynamics analysis of Neosetophomone B (NSP‐B) and AKT complex. (A) Showing the 2D interaction of NSP‐B with AKT. (B) Showing the 3D interaction of NSP‐B with AKT. (C) Showing the dynamic stability of the complex as a root mean square deviation (RMSD) value and (D) showing the compactness of the complex as a radius of gyration (Rg) value. (E) Showing the average hydrogen bonds of the complex. (F) Showing the residual fluctuation of the complex as root mean square fluctuation value. AKT, protein kinase B.

Our findings demonstrated a notable inactivation of AKT in both cell lines following NSP‐B treatment, as shown in Figure 7A. To further analyse the mechanism of this inactivation, we performed the molecular docking of NSP‐B with AKT. The molecular docking was performed by defining the amino acids residues reported previously.^52^ The docking analysis of NSP‐B and AKT complex reported a docking score of −6.767 kcal/mol with three hydrogen bonds in the complex. The key hotspot residues Glu191, Glu278 and Asp292 were involved in hydrogen bond formation. Interestingly, these particular residues have been reported previously as essential components of the active site of AKT.^52^ The consistency of these findings emphasizes the reliability and relevance of the docking results. The involvement of Glu191, Glu278 and Asp292 in hydrogen bond formation suggests that NSP‐ B establishes specific and favourable interactions with the active site of AKT. This insight underscores the binding potential of NSP‐B towards AKT, providing valuable information for understanding the molecular interactions involved. The 2D and 3D pattern of NSP‐B‐AKT is shown in Figure 7A,B.

### 3.7 Dynamic stability and binding free energy analysis of NSP‐B–AKT complex

In order to assess whether NSp‐B maintains its stability throughout the simulation, the RMSD values for the trajectories over time were computed.^53^, ^54^ The complex stabilized at 1.5 Å, exhibiting consistent stability without major deviation during the entire simulation period. The average RMSD for this complex, measured at 2.0 Å, displayed a consistently straight line pattern, indicative of stable dynamics maintained throughout the simulation (Figure 7C). The radius of gyration (Rg) serves as a metric indicating the compactness or structural stability of a ligand‐protein complex. The NSp‐B‐AKT complex consistently maintained a compact topology throughout the simulation, with an average Rg value of 20.1 Å (Figure 7D). The NSp‐B‐AKT complex displayed an average of 145 hydrogen bonds, as shown in Figure 7E. In the realm of molecular biology and structural bioinformatics, the RMSF calculation serves as a valuable tool for studying protein‐drug interactions.^55^, ^56^ It provides critical insights into the dynamic behaviour of proteins, particularly in understanding their interactions with drugs.^57^, ^58^ Figure 7F illustrates that the NSp‐B‐AKT complex achieved a notable equilibrium state among its residues, with an average RMSF value of 1 Å. Furthermore, to analyse the real binding strength of NSp‐B with AKT we calculated the binding free energy by using the MM/generalized Born surface area approach. The NSp‐B and AKT complex revealed a Van der Waals energy of −39.64 ± 0.82 kcal/mol and an electrostatic energy of −11.63 ± 0.39 kcal/mol, emphasizing the considerable impact of electrostatic forces on the overall binding energy. However, the total bindig free energy of NSp‐B‐AKT complex was recorded to be −29.41 ± 0.74 kcal/mol. In conclusion, the comprehensive pharmacological evaluation of NSp‐B in its complex with AKT reveals promising attributes for potential therapeutic applications in T‐ALL and provides a strong foundation for further exploration of its therapeutic potential.

## 4 DISCUSSION

The emerging popularity and research focus on natural compounds as potential anti‐cancer agents have opened new vistas in the fight against malignant tumours.^59^, ^60^ These bioactive substances have shown promise in cancer prevention and treatment by interacting with various signalling molecules and pathways.^61^, ^62^ Additionally, there is increasing evidence that natural compounds can enhance the effectiveness of conventional chemotherapy drugs.^6^ A key characteristic of effective anti‐cancer agents is their capacity to induce apoptosis in malignant cells, often by targeting signal transduction pathways linked to the inhibition of cancer cell growth.^63^ NSP‐B, a meroterpenoid fungal secondary metabolite isolated from Neosetophoma sp., has previously been demonstrated to induce apoptosis in various cancer cell lines.^8^, ^24^, ^25^, ^26^

Our study's findings corroborate that NSP‐B treatment in T‐ALL cells significantly inhibits cell growth and induces apoptosis. Apoptosis is a complex, multi‐step process involving numerous genes responsible for its regulation and execution.^64^

Activated AKT has been associated with poor disease‐free survival in breast and non‐small‐cell lung cancers.^65^ Inhibitors like LY294002 have been shown to effectively induce apoptosis in diffuse B‐cell lymphoma by targeting the PI3K/AKT pathway.^66^ Our data confirm the frequent activation of PI3K in T‐ALL cell lines, evidenced by the constitutive phosphorylation of its downstream substrates, including AKT and GSK3. NSP‐B treatment correspondingly inhibits AKT and GSK3 signalling pathways. PI3K‐mediated AKT activity, present in both the mitochondrial matrix and membranes, is crucial for maintaining mitochondrial membrane integrity.^67^, ^68^ The dysfunction of this membrane, indicated by the loss of mitochondrial membrane potential, often results from imbalances in BCL2 and Bax protein levels. The Bax/Bcl2 ratio increase can lead to mitochondrial‐mediated apoptosis by many anti‐cancer agents.^69^ In our study, NSP‐B treatment in T‐ALL cells suppressed BCL2 expression while increasing Bax levels, suggesting that NSP‐B induces apoptosis through mitochondrial signalling pathways.

mTOR, a central regulator in cell growth and survival, plays a pivotal role in various human cancers. In T‐ALL, mTOR is frequently dysregulated, contributing to malignant cell proliferation and survival. Our data demonstrate that NSP‐B treatment significantly impacts mTOR signalling. Specifically, NSP‐B leads to the dephosphorylation of mTOR at Ser2448, a critical site for its activation, thus indicating a suppression of mTOR activity. This inactivation is accompanied by reduced expression of translational repressors like 4E‐BP1, a direct target of mTOR signalling. Furthermore, flow cytometric analysis revealed that NSP‐B treatment markedly diminishes the phosphorylation levels of S6, a downstream target of mTORC1, signifying an overall downregulation of mTOR signalling in T‐ALL cells. These findings suggest that NSP‐B's anti‐cancer activity in T‐ALL might be partially attributed to its ability to disrupt mTOR‐mediated pathways, offering a novel therapeutic angle in T‐ALL treatment.

The role of IAPs in cancer cell resistance to apoptosis is well‐documented.^70^, ^71^ These proteins, by inhibiting caspase activity, protect cells from apoptosis induced by various agents.^72^ AKT is known to regulate the expression of genes associated with cell survival, including IAPs.^73^ In our study, T‐ALL cell lines exhibited the presence of IAPs, such as XIAP, cIAP1 and cIAP2. Significantly, NSP‐B treatment reduced the expression of these IAPs, suggesting that downregulation of IAPs may be crucial in initiating the caspase cascade signalling during NSP‐B‐induced apoptosis.

Our experiments with primary T‐cell PHA‐blasts, which mimic T‐ALL, demonstrate that NSP‐B effectively induces cell death in activated cells and inhibits cell proliferation through G0/G1 cell cycle arrest. These findings indicate that NSP‐B can selectively target actively proliferating or transformed T cells. Notably, the lack of similar effects on unstimulated primary T cells treated with NSP‐B suggests that this compound has a degree of selectivity, preferentially acting on T‐ALL cells and activated T cells without affecting normal, quiescent T cells.

Our molecular docking analysis revealed a significant interaction between NSP‐B and AKT, with a docking score of −6.767 kcal/mol, suggesting a strong binding affinity. This interaction involves key residues in AKT's active site, which is crucial for its kinase activity. Such binding specificity of NSP‐B towards AKT underscores its potential as a targeted therapeutic agent. Furthermore, dynamic simulation studies showed that the NSP‐B‐AKT complex maintains consistent stability, demonstrated by an average RMSD of 2.0 Å and a compact topology throughout the simulation. The complex also exhibited a substantial number of hydrogen bonds, further confirming its stability. These computational insights complement our biochemical findings, suggesting that NSP‐B's inhibitory effects on the AKT pathway in T‐ALL may stem from its direct interaction and binding stability with AKT.

In summary, our findings on the molecular docking and dynamic stability of NSP‐B with AKT, combined with its impact on key signalling pathways in T‐ALL cells, provide a deeper understanding of its mechanism of action. These insights strengthen the potential of NSP‐B as a novel therapeutic agent in T‐ALL treatment, offering a multifaceted approach by targeting both the PI3K/AKT and mTOR pathways and influencing apoptotic regulators.

## AUTHOR CONTRIBUTIONS

**Shilpa Kuttikrishnan**: Data curation; Formal analysis; methodology; validation; writing—original draft; writing—review and editing. **Abdul W. Ansari**: Conceptualization; data curation; formal analysis; investigation; methodology; writing—review and editing. **Muhammad Suleman**: Conceptualization; data curation; formal analysis; investigation; methodology; writing—review and editing. **Fareed Ahmad**: Data curation; formal analysis; methodology; writing—review and editing. **Kirti S. Prabhu**: Data curation; formal analysis; methodology; project administration; resources. **Tamam El‐Elimat**: data Curation; resources; writing—review and editing. **Feras Q. Alali**: Data curation; resources; writing—review and editing. **Ammira S. Al Shabeeb Akil**: Data curation; resources; writing—review and editing. **Ajaz A. Bhat**: Data curation; resources; writing—review and editing. **Maysaloun Merhi**: Data curation; resources; writing—review and editing. **Said Dermime**: Data curation; resources; writing—review and editing. **Martin Steinhoff**: Conceptualization; resources; writing—review and editing. **Shahab Uddin**: Conceptualization; supervision; data curation; formal analysis; methodology; validation; writing—original draft; writing—review and editing.

## FUNDING INFORMATION

This work was supported by grants funded by the Medical Research Center (MRC), Hamad Medical Corporation, Doha, Qatar (MRC‐01‐21‐301).

## CONFLICT OF INTEREST STATEMENT

The authors do not have any conflict of interest to declare.

## INFORMED CONSENT/PATIENT CONSENT

Prior written informed consents were obtained from each participant and study was conducted according to Helsinki Declaration.

## Data Availability

The data that support the findings of this study are available from the corresponding author upon reasonable request.
